# Systemic network analysis identifies XIAP and IκBα as potential drug targets in TRAIL resistant BRAF mutated melanoma

**DOI:** 10.1038/s41540-018-0075-y

**Published:** 2018-11-05

**Authors:** Greta Del Mistro, Philippe Lucarelli, Ines Müller, Sébastien De Landtsheer, Anna Zinoveva, Meike Hutt, Martin Siegemund, Roland E. Kontermann, Stefan Beissert, Thomas Sauter, Dagmar Kulms

**Affiliations:** 1 Experimental Dermatology, Department of Dermatology, TU-Dresden, Dresden, 01307 Germany; 2 Center of Regenerative Therapies Dresden, TU-Dresden, Dresden, 01307 Germany; 3 Systems Biology, Life Science Research Unit, University of Luxembourg, Belvaux, 4367 Luxembourg; 4 Institute of Cell Biology and Immunology, University of Stuttgart, Stuttgart, 70569 Germany; 5 Stuttgart Research Center Systems Biology, University of Stuttgart, Stuttgart, 70569 Germany

## Abstract

Metastatic melanoma remains a life-threatening disease because most tumors develop resistance to targeted kinase inhibitors thereby regaining tumorigenic capacity. We show the 2nd generation hexavalent TRAIL receptor-targeted agonist IZI1551 to induce pronounced apoptotic cell death in *mut*BRAF melanoma cells. Aiming to identify molecular changes that may confer IZI1551 resistance we combined Dynamic Bayesian Network modelling with a sophisticated regularization strategy resulting in sparse and context-sensitive networks and show the performance of this strategy in the detection of cell line-specific deregulations of a signalling network. Comparing IZI1551-sensitive to IZI1551-resistant melanoma cells the model accurately and correctly predicted activation of NFκB in concert with upregulation of the anti-apoptotic protein XIAP as the key mediator of IZI1551 resistance. Thus, the incorporation of multiple regularization functions in logical network optimization may provide a promising avenue to assess the effects of drug combinations and to identify responders to selected combination therapies.

## Systems Biology and Cancer: Network analysis for TRAIL resistance in Melanoma

Even though targeted kinase inhibitors have been invented to mutation-specifically fight melanoma, most patients present with initial or acquired resistance and suffer from tumor relapse. A multidisciplinary team lead by Dagmar Kulms at the Technical University of Dresden, has developed a predictive Dynamic Bayesian Network model that is able to predict alternative targets for melanoma treatment. Comparing melanoma cells being sensitive or resistant to death receptor induced apoptosis by a second-generation TRAIL agonist the model predicted molecular signatures of pro-survival/anti-apoptotic responses in a dynamic manner. They show that TRAIL holds a great potential of success for targeted cancer therapy in the near future, and that the incorporation of multiple regularization functions in logical networks may provide a promising avenue to assess the effects of drug combinations for personalized medicine.

## Introduction

Dysregulation of two major mitogen-activated pathways (RAS-RAF-MEK-ERK and PI3K-AKT-PTEN) are key drivers of melanoma development and progression,^1^ with 66% of patients expressing a constitutive active mutant of the MAP (mitogen-activated protein)-kinase BRAF (*mut*BRAF, V600D or V600E).^2^ The initial response rates of patients to first-line therapy with targeted *mut*BRAF inhibitors dabrafenib or vemurafenib is almost 100%, however about 70% of patients acquire resistance to the treatment within one year.^3,4^ Accordingly, downstream inhibition of the MAP-kinase MEK with e.g., trametinib is used as a second-line therapy or even initially combined with *mut*BRAF-inhibitors.^5,6^ Still, the prognosis for patients with metastatic melanoma remains particularly poor and is mostly associated with high tumor relapse rates.^1^^[,3,7^

Therefore, alternative treatment options are demanded as first or second line therapy to overcome acquired resistance. In this context, cell death induction by the tumor-selective death ligand TRAIL (Tumor necrosis factor-Related Apoptosis-Inducing Ligand) might serve as an alternative treatment option. Unfortunately, melanoma cells were shown to stay largely resistant against conventional TRAIL treatment.^8,9^ Importantly, conventional trimeric TRAIL and receptor-agonistic antibodies as single agents failed in clinical trials, due to the limited therapeutic activity in patients.^10^ To overcome this therapeutic limitation we have developed novel second-generation TRAIL receptor-targeted agonists, with increased bioactivity enhancing the cytotoxic capacity towards cancer cells. These fully human TRAIL-Fc-fusion proteins consist of two single-chain TRAIL molecules fused covalently to the Fc-part of human IgG, forming a potent hexameric TRAIL-receptor agonist (IZI1551). Systemic administration of IZI1551 in mice xenograft models resulted in a potent antitumoral activity with improved pharmacokinetic properties showing no side effects.^11,12^

However, both MAPK signalling as well as TRAIL receptor activation can lead to the activation of the transcription factor NFκB^10,13–15^ which may impair the therapeutic outcome due to upregulation of survival genes. To take this sensitive balance between pro-apoptotic and anti-apoptotic signalling into account, and to explore novel treatment options, a holistic understanding of the signal transduction network within melanoma cells is a prerequisite.

To assess the relevance of individual interactions within the signal transduction network of melanoma cells, we applied Dynamic Bayesian Network (DBN) modelling, which allows to efficiently contextualize and analyze logical networks. The parameters of DBN models can be estimated using quantitative (quasi) steady-state protein data, thereby for example allowing comparisons between cell types,^16^ here of therapy-responsive and therapy-resistant melanoma cell lines. Large-scale DBN modelling is feasible with the recently published FALCON toolbox,^17^ a Matlab-based framework designed for computational performance, and comprising a wide range of systems-level analyses. Furthermore, additional constrains on the parameter set can be included in the optimization problem in the form of biased estimators. Such regularized objective functions are frequently used to balance goodness-of-fit with existing prior assumptions.^18^ Two desired properties of the parameter values of a meta-model encompassing the different cell types can be formulated. Firstly, it is expected that the phenotypic differences between the cell lines are due to a limited number of molecular changes, and that therefore most cellular processes are identically parametrized across both cell types. Secondly, the final model should be as sparse as possible by focusing on the most essential interactions, to increase its predictive power and to facilitate interpretation.

In order to identify the molecular changes between TRAIL-sensitive melanoma cells compared to melanoma cells that have acquired resistance to TRAIL we used a mixed regularization scheme incorporating these two assumptions within the FALCON toolbox to estimate parameter values for the two cell types and discover the most significant changes that may confer therapy resistance.

## Results

### Hexavalent TRAIL receptor agonist IZI1551 is superior in killing *mut*BRAF melanoma cells to conventional TRAIL or specific MAP-kinase inhibitors

**Fig. 1:**
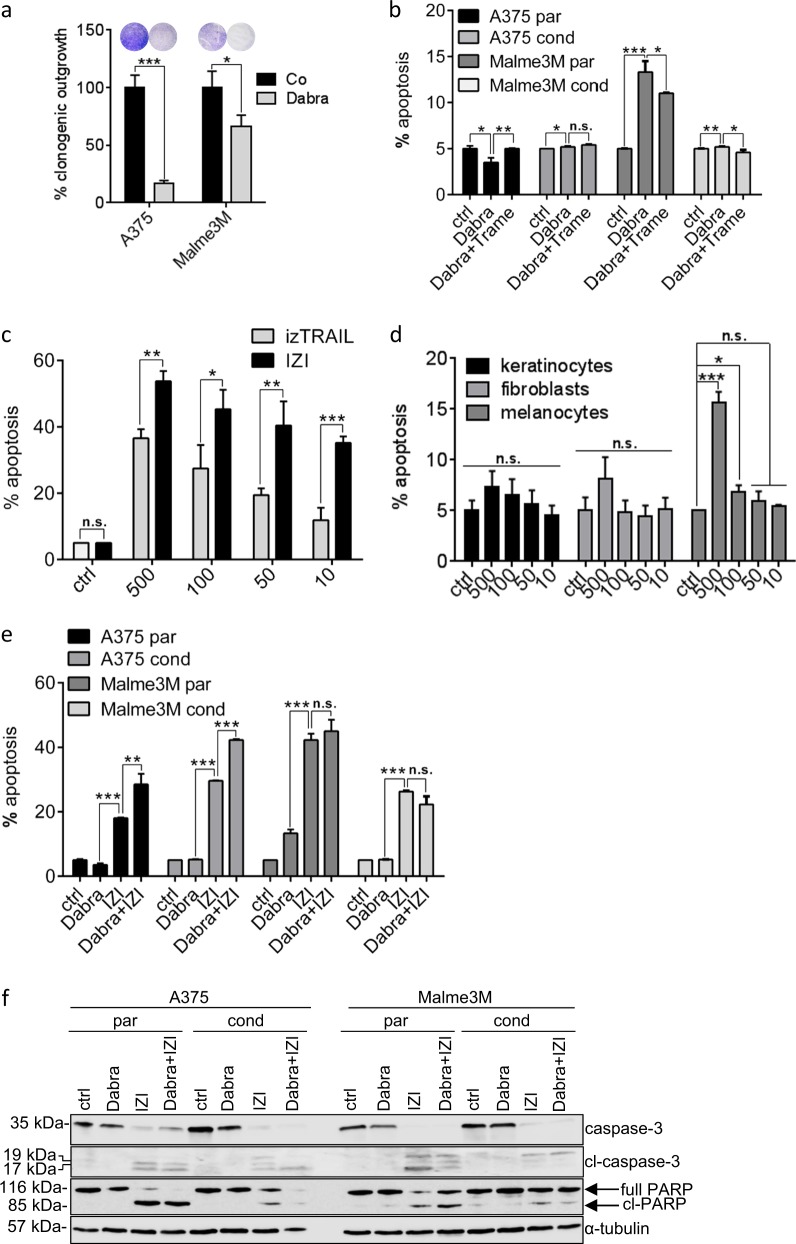
IZI1551 is superior in killing melanoma cells than TRAIL or specific MAP kinase inhibitors. **a** Clonogenic outgrowth of *mut*BRAF A375 and Malme3M melanoma cells treated with dabrafenib (Dabra; 10 µM) for 8 days was compared to untreated cells (*p ≤ 0.05; ***p ≤ 0.001). **b** Parental and dabrafenib-conditioned melanoma cell lines A375 and Malme3M were treated with dabrafenib (Dabra; 10 µM) alone or in combination with trametinib (Trame; 1 µM). After 48 h apoptosis was determined using a Cell Death Detection ELISA (CDDE) (*p ≤ 0.05; **p ≤ 0.01; ***p ≤ 0.001; n.s. = not significant). **c** A375 melanoma cells were treated with increasing doses of izTRAIL or IZI1551 as indicated (ng/ml). After 24 h apoptosis was determined using a CDDE (*p ≤ 0.05; **p ≤ 0.01; ***p ≤ 0.001; n.s. = not significant). **d** The same dose kinetics of IZI1551 as in (**c**) was applied to primary human keratinocytes, fibroblasts and melanocytes. After 24 h apoptosis was determined using a CDDE (*p ≤ 0.05; ***p ≤ 0.001; n.s. = not significant). **e** Parental (par) and dabrafenib-conditioned (cond) melanoma cell lines A375 and Malme3M were treated with dabrafenib (Dabra; 10 µM) or IZI1551 (IZI; 50 ng/ml) alone or in combination. After 24 h of IZI1551 and 48 h of dabrafenib treatment apoptosis was determined using a CDDE (**p ≤ 0.01; ***p ≤ 0.001; n.s. = not significant) and **f** monitored by Western-blot analysis using antibodies against caspase-3 and PARP. α-tubulin served as loading control. For CDDE and clonogenic assay, the mean ± SD of three independently performed experiments is shown. WBs represent one out of three independently performed experiments

Once diagnosed, the first-line therapy of *mut*BRAF melanoma includes administration of specific kinase inhibitors like vemurafenib or dabrafenib. Accordingly, treatment of two *mut*BRAF melanoma cell lines A375 and Malme3M with dabrafenib (Dabra) reduced clonogenic outgrowth, indicating growth inhibition to occur in response to *mut*BRAF inhibition (Fig. 1a and Figure S1). However, active cell death induction remained largely absent in response to dabrafenib alone, as well as in combination with the MEK inhibitor trametinib (Trame), as used as second line therapy for patients who have acquired resistance against *mut*BRAF inhibitors (Fig. 1b). To mimic dabrafenib resistance we conditioned melanoma cells to a sub-lethal dose of dabrafenib (1 µM) over a period of six months. Neither conditioned, nor non-conditioned, parental cells responded with significant cell death induction to the combination of two downstream MAPK pathway inhibitors (Fig. 1b), implying that additional cell death induction might be superior to MEK-inhibition in (re-)sensitizing *mut*BRAF melanoma.

Consequently, conventional trimeric isoleucine-zipper linked TRAIL (izTRAIL) induced moderate apoptotic cell death in A375 melanoma cells while hexavalent scTRAIL-Fc fusion protein (IZI1551) even showed increased cytotoxic activity (Fig. 1c). IZI1551-induced cytotoxicity was shown to be largely tumor-selective, as it spared primary keratinocytes, fibroblasts and melanocytes of the skin from apoptotic cell death induction (Fig. 1d). Moreover, IZI1551 (IZI) was shown to be significantly more potent in actually killing parental as well as in re-sensitizing dabrafenib-conditioned *mut*BRAF melanoma cells than the specific *mut*BRAF inhibitor dabrafenib (Dabra) (Fig. 1e). Accordingly, apoptotic cell death induction through cleavage of the executioner caspase-3 as well as its substrate PARP was exclusively evident in both, parental and conditioned cells upon treatment with IZI alone or in combination with dabrafenib (Fig. 1f).

### Monitoring IZI1551 susceptibility using mathematical modelling

**Fig. 2:**
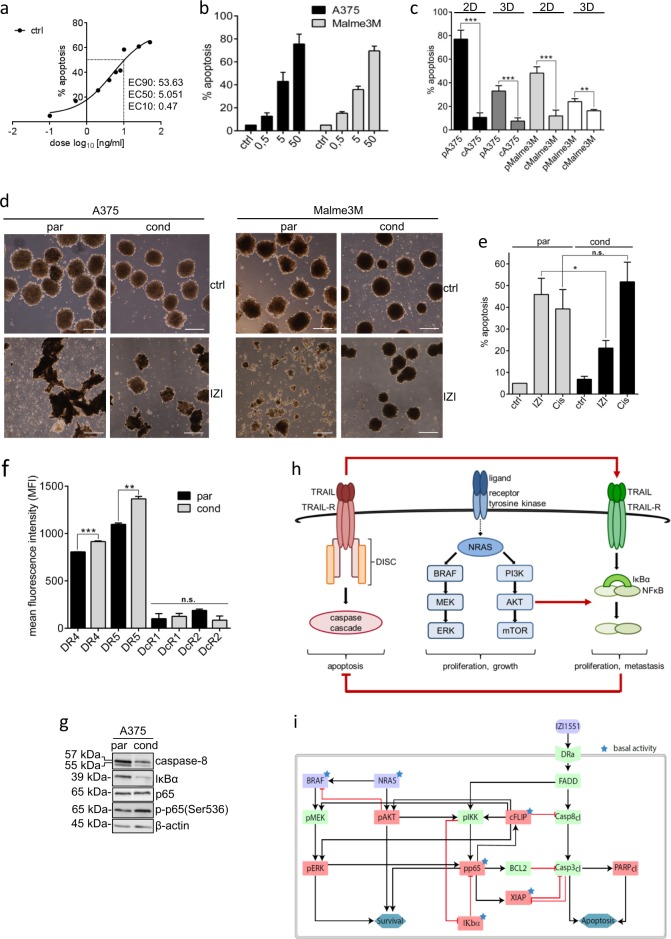
Monitoring IZI1551 susceptibility using mathematical modelling. **a** Dose response curve of nine different IZI1551 concentrations to determine the EC50 concentration. **b** A375 and Malme3M melanoma cells were treated with increasing IZI1551 doses (0.5; 5; and 50 ng/ml) and apoptosis determined 24 h later in a CDDE. **c** Parental and IZI1551-conditioned A375 and Malme3M cells in 2D cell culture and in 3D spheroid culture, respectively, were treated with IZI1551 (50 ng/ml). After 24 h apoptosis was determined using a CDDE (**p ≤ 0.01; ***p ≤ 0.001), and **d** monitored by transmission microscopy of 3D spheroids. Scale bar = 250 µm. **e** Parental and IZI1551-conditioned A375 cells were treated with IZI1551 (50 ng/ml) or cisplatin (30 µM). After 24 h apoptosis was determined using a CDDE (*p ≤ 0.05; n.s. = not significant). **f** Surface expression level of TRAIL receptors 1 (DR4) and 2 (DR5) and decoy receptors 3 (DcR1) and 4 (DcR2) of parental (par) and conditioned (cond) A375 cells was scored by FACS analysis (**p ≤ 0.01; ***p ≤ 0.005; n.s. = not significant). **g** The expression level of caspase-8, IκBα, NFκB(p65) and phosphorylated-p65(Ser536) in untreated parental and IZI-conditioned A375 cells was monitored by Western-blot analysis. β-actin served as loading control. One representative Western-blot out of three independently performed experiments is shown (for two more replicates, see Figure S2a). **h** Schematic overview of MAPK-dependent, TRAIL-induced pro-apoptotic and NFκB-driven anti-apoptotic signal transduction pathways. **i** Topology of the Dynamic Bayesian Network (DBN) model of the signal transduction pathways. Black arrows indicate the activation, red arrows the inhibition of target proteins (purple nodes = model inputs, red nodes = measured proteins, green nodes = not measured proteins, blue nodes = functional measurements, asterisks = constitutively active proteins). For CDDE and flow cytometry analysis the mean ± SD of three independently performed experiments is shown

In order to investigate the potential of IZI1551 as an alternative treatment option for malignant melanoma, we aimed at identifying molecular changes and switches that might occur during acquired TRAIL resistance, and thus conditioned *mut*BRAF A375 and Malme3M melanoma cells to the EC50 IZI1551 dose (5 ng/ml) for 6 months (Fig. 2a, b and Table S1). Compared to parental cells (pA375; pMalme3M), conditioned cells (cA375; cMalme3M) stayed largely resistant to treatment with a lethal dose of IZI1551 (50 ng/ml, Fig. 2c, compare Fig. 2b and Table S1). The overall response to IZI1551 was lower in parental 3D spheroid culture, mimicking the architecture of tumor metastasis *in vivo*,^19–21^ as compared to regular 2D cell culture, and remained largely absent in conditioned 3D spheroids (Fig. 2c). Accordingly, only parental 3D spheroids were shown to be disrupted due to cell death induction 24 h after treatment with IZI1551 (Fig. 2d).

Interestingly, IZI1551-resistant cA375 cells had not generally lost the capacity to induce cell death, since they could still respond to cisplatin treatment with apoptosis induction (Fig. 2e). Neither downregulation of apoptosis-inducing TRAIL receptors 1 (DR4) and 2 (DR5) nor upregulation of TRAIL-decoy receptors DcR1 or DcR2 was evident to confer IZI1551 resistance in conditioned melanoma cells (Fig. 2f). The major dysregulation identified at the molecular level displayed downregulation of the initiator caspase-8 (Fig. 2g, Figure S2a). This is due to the conditioning process in which only those cells survive the constant exposure to 5 ng/ml TRAIL agonist which express only low levels of caspase-8. Under these conditions, caspase-8 seems to serve a non-catalytic scaffold function, leading to cytokine production via NFκB activation, instead of cell death.^22,23^ Along this line, also the protein level of the NFκB inhibitor IκBα was shown to be reduced, accounting for constitutive activation of the transcription factor NFκB, being also evident by enhanced phosphorylation of its p65 subunit (Fig. 2g). It therefore appeared that melanoma cells surviving TRAIL receptor activation selectively reduced the apoptotic signal transduction by downregulating the receptor-associated initiator caspase-8 and at the same time activated NFκB, which is usually associated with upregulation of anti-apoptotic genes.^24^

For the mathematical modelling based network analysis we therefore focused on the integration of three signal transduction pathways that may influence melanoma progression and treatment: MAPK signaling—as frequently dysregulated in melanoma, extrinsic death receptor-driven apoptosis, and alternative death receptor-driven anti-apoptotic NFκB activation (Fig. 2h). In order to disentangle the complexity of melanoma resistance, we established a DBN model^17^ comprising the selected signal transduction pathways as well as their crosstalk to precisely identify the most sensitive nodes within this signal transduction network that may serve as druggable targets. The network topology was assembled from literature and public databases (Metacore and Ingenuity), and comprised 19 nodes and 29 parameters. We calibrated models independently for each cell type (Fig. 2i) with quantitative protein expression and activation data of MAPK members AKT and ERK, the pro-apoptotic protein PARP, and anti-apoptotic proteins including IκBα, NFκB (p65), FLIP, and XIAP derived from immunoblotting of unstimulated and stimulated parental versus conditioned A375 cells (Figure S2b and S2c).

We deliberately established the DBN model exclusively on data derived from parental and conditioned A375 cells, intending to utilize Malme3M cells to validate the predictive power of the model retrospectively.

### Accurate modelling requires apoptotic proteins in parental but mostly NFκB-driven anti-apoptotic proteins in conditioned cells

**Fig. 3:**
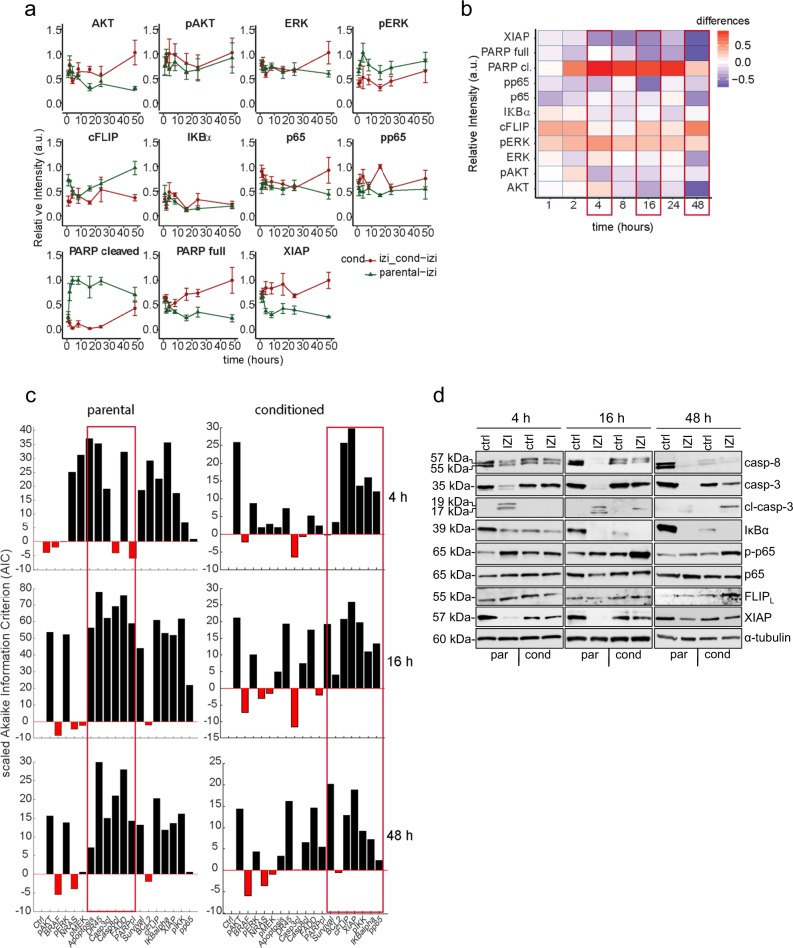
Accurate modelling requires apoptotic proteins in parental but mostly NFκB-driven anti-apoptotic proteins in conditioned cells. **a** The expression pattern of AKT, ERK, FLIP, XIAP, IκBα, NFκB(p65), and PARP proteins were analysed by quantitative immunoblotting in whole-cell lysates of parental and IZI1551-conditioned A375 cells stimulated with IZI1551 (50 ng/ml) for 1, 2, 4, 8, 16, 24, and 48 h. The corresponding protein values were normalized between 0 and 1. Error bars represent the SEM with *n* = 5. **b** Heatmap representing the time-dependent differences between protein expression in parental and IZI1551-conditioned A375 cells. **c** Systematic in silico knock-out analysis of each individual protein at 4, 16, and 48 h. The Akaike Information Criterion (AIC) for the reference model is scaled to 0. Parameters playing an important role in the optimization are displayed in black (parameter > 0). Parameters having no effect on the model optimization are displayed in red (parameter < 0). **d** Parental and IZI1551-conditioned A375 cells were treated with IZI1551 (50 ng/ml). After 4, 16, and 48 h the status of caspase-8, caspase-3, IκBα, p-p65(S536), p65, FLIP_L_, and XIAP was determined by Western-blot analysis. GAPDH served as loading control. One out of five independently performed experiments is shown

Acquired TRAIL resistance during conditioning of cells to IZI1551 caused severe modifications in expression levels of anti-apoptotic proteins XIAP and FLIP, as well as in the activation status of pro-survival proteins NFκB, IκBα, AKT, ERK, and pro-apoptotic protein PARP over time (1, 2, 4, 8, 16, 24, 48 h) (Fig. 3a and Figure S2a and the heatmap Figure S2b which includes the values of the untreated and treated samples). We analyzed the differences in normalized protein expression in parental and IZI1551-conditioned A375 cells for each time point. The largest overall differences between the profiles of parental and IZI1551-conditioned A375 cells were observed at the three different time points that might be referred to as: initiation phase (4 h), execution phase (16 h), and adaption phase (48 h) (Fig. 3b). To analyze the network modularity within these three phases, we performed systematic in silico protein knock-out experiments in the parental and IZI1551-conditioned cells at 4, 16 and 48 h. We used the Akaike Information Criterion (AIC) as selection criteria to verify if the selective removal of each individual node can be compensated by the network. Based on this analysis mostly pro-apoptotic proteins were shown to play an essential role in the execution and adaption phase of parental cells in response to IZI1551 treatment. In contrast, pro-apoptotic proteins only played a minor role in IZI1551-treated conditioned cells, because right from the initiation phase and through the execution and adaptation phases NFκB-dependent anti-apoptotic proteins were shown to be the most indispensable for accurate modelling of the system (Fig. 3c).

In accordance with the results from the in silico knock out, Western-blot analysis confirmed lack of pro-apoptotic caspase-3 processing and concomitant NFκB activation to be key characteristics of conditioned A375 cells in response to IZI1551 treatment compared to parental cells (Fig. 3d). As a consequence IZI1551-induced depletion of anti-apoptotic proteins FLIP and XIAP was fully compensated most likely due to upregulation of these NFκB-dependent genes (Fig. 3d).^8,9^ Immunoblotting in concert with the mathematical model analysis strongly implied the balance at the TRAIL receptor of IZI1551 conditioned cells to switch from pro-apoptotic caspase-dependent signal transduction to NFκB-driven anti-apoptotic signaling, which finally may confer TRAIL resistance.

Considering the overall network sensitivity upon in silico knock out of each node, the execution phase (16 h) seems to represent the time-point of maximal vulnerability to systems perturbation between parental and conditioned A375 cells. Based on the analysis and taking into account the fact that the initiation phase (4 h) might not represent the steady-state of the signaling network, while in the adaption phase (48 h), the effects of transcriptional regulation might already be too large and might alter the wiring of the signaling machinery, we selected the 16 h time-point for further analysis.

### Model analysis predicts that dysregulated XIAP and IκBα drive IZI1551 resistance in melanoma

In order to integrate the experimental data into a coherent picture and to gain a systems-level understanding of the signal transduction network affected by IZI1551 conditioning, we implemented different regularization algorithms in the FALCON toolbox to identify the cell type-specific parameters. We therefore combined two regularization methods. The partial-norm (L_1/2_) regularization method optimizes identical models for multiple series of experimental conditions in parallel and allows discovering those parts of the network that are active or inactive between cell lines, resulting in pruning of inactive edges within the experiments. The grouped L_1_ regularization for each interaction focuses on the differences between parental and conditioned cells and tends to reduce the model size by assigning the same parameter value for both cell types for a given interaction. Here, we combined both methods to identify the minimal network structure and uncover cell type-specific differences.

**Fig. 4:**
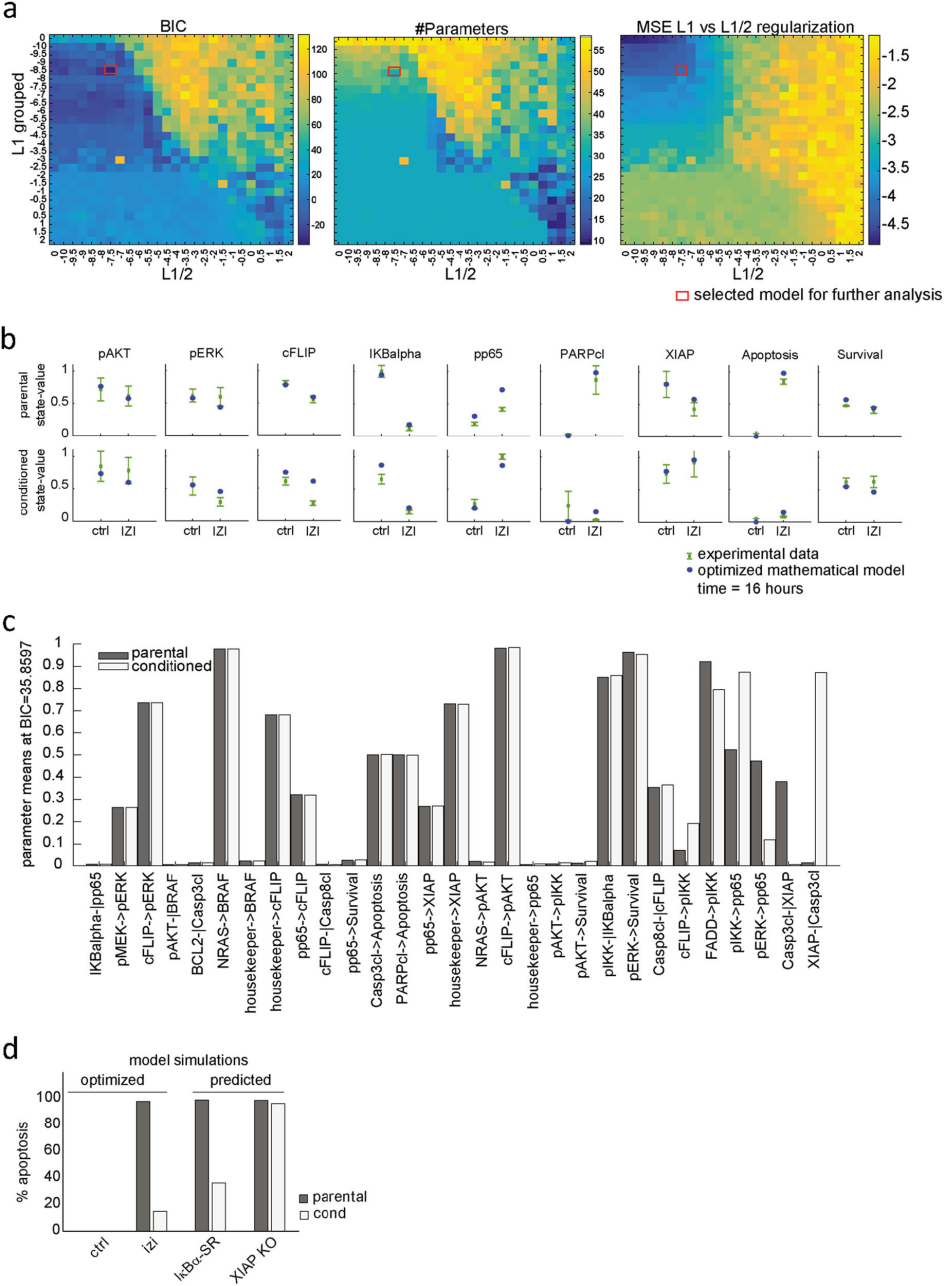
Model analysis predicts that dysregulated XIAP and IκBα drive IZI1551 resistance in melanoma. **a** Combined optimization of the grouped L1 and L1/2 regularization algorithms. BIC: The Bayesian Information Criterion (BIC) was used to obtain the best model structure. #Parameters: The number of non-null parameters for each model variant. MSE: Logarithm of the mean squared error indicating the quality of the fit compared to the experimental data. *X*-axis (left to right): increasing the L1/2 regularization. *Y*-axis (top to bottom): increasing the L1 grouped regularization. Tiles: blue the smallest (best) and yellow the largest (worst) values for the BIC, #Parameters and the MSE L1 vs L1/2 regularization. The red box indicates the model with the best BIC. **b** Comparison of the simulated node activity obtained in the optimal model and the protein quantification for the parental and IZI1551-conditioned A375 cells. Blue dots represent the simulated node activity; green dots the average of five measurements with standard error of the mean. **c** Optimal parameter values for both cell lines. The parameters are sorted from the lowest (left) to the highest (right) difference in parameter values between cell lines. **d** Model predictions based on the optimized mathematical model simulating the effect of the IκBα super repressor (IκBα-SR) and XIAP knock-out (XIAP KO) on % apoptosis being induced upon IZI1551 treatment of parental and IZI1551-conditioned A375 cells

To identify the minimal set of reactions in the network (L_1/2_) as well as the minimal number of parameters between cell lines (L_1_ groups), we screened values for the regularization strengths from 10E-10 to 10E2 by half-log steps, thus giving 25 different values to test, plus 0. We performed the optimization for each combination of these values, thus optimizing in total 26 L_1/2_ × 26 L_1_-grouped regularization strengths = 676 models. Out of 676 different model structures investigated, we identified the optimal network structure based on the Bayesian Information Criterion (BIC) (Fig. 4a; BIC: red box).^25^ The optimal network (Fig. 4a; #Parameters) contained 29 parameters comprising 19 parameters with equal values for both cell types, four reactions equal to 0, and six cell type-specific reactions, while the initial network contained 58 non-zero parameters (2 cell lines × 29 parameters). The goodness-of-fit of the reduced model was assessed by the mean squared error (Fig. 4a; MSE: red box). The total runtime of the experiment was 6.2 h for assessing the 676 model variants, i.e., ~13 s per individual model. The reduced mathematical model with only 31 non-zero parameters for both cell lines was shown to be able to describe the experimental data (Fig. 4b) to a similar extent as the complete mathematical model which is considering different parameters for both cell types. These results show that our modeling pipeline is able to identify major putative differences in parameter values between parental and conditioned cells (Table S2).

The relevance of these differences can be further analyzed by parameter comparison, as displayed by parameter values sorted by increasing difference between both cell types (Fig. 4c). This allows estimating if a reaction is cell type-specific and/or essential. The reactions which strongly differ between both cell types (Fig. 4c, right columns) are mainly linked to the NFκB activating and apoptosis-inducing pathways (Table S2). Reactions from the anti-apoptotic proteins FLIP (FLIP -|Casp8cl) and also BCL2 (BCL2 -|Casp3cl) were close to 0 in the parental and conditioned cells, meaning that the inhibition strength of both proteins would not be enough to inhibit apoptosis. The strongest difference between parameter values can be observed in the reaction XIAP -| Casp3cl, absent in parental cells (*k* = 0.0135) but highly active in conditioned cells (*k* = 0.8715). Considering these modelling results, one would expect that IZI1551-conditioned cells upregulated the apoptosis inhibitor XIAP to acquire resistance to the treatment. Accordingly, the regularized model predicts IκB super repressor (IκB-SR) to partially, and XIAP knockout to fully re-sensitize conditioned cells (Fig. 4d).

### DBN modelling correctly predicts melanoma cell re-sensitization to IZI1551 by targeting NFκB or XIAP

**Fig. 5:**
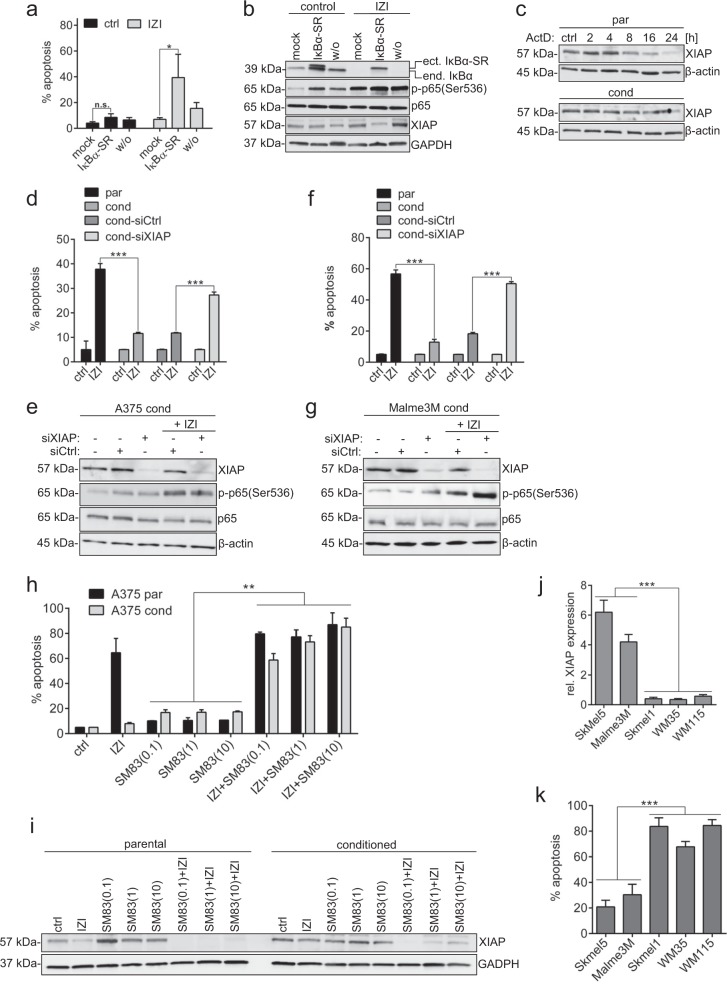
Depletion of XIAP re-sensitizes melanoma cells to IZI1551. **a** IZI1551-conditioned A375 melanoma cells were transiently transfected with an IκBα super repressor (IκBα-SR) or the empty vector (mock) and treated with IZI1551 (50 ng/ml). After 16 h apoptosis was determined using a CDDE (*p ≤ 0.05; n.s. = not significant) and **b** the status of IκBα, p-p65(Ser536), p65, and XIAP monitored by Western-blot analysis. GAPDH served as loading control. **c** Transcription was inhibited in parental and IZI1551-conditioned A375 cells by addition of Actinomycin D (ActD, 1 µM) for the indicated time points. Protein level of XIAP was monitored by Western-blot analysis. GAPDH served as loading control. **d** XIAP was depleted from parental and IZI1551-conditioned A375 and **f** Malme3M cells using RNAi for 72 h. Sixteen hours after treatment with IZI1551 (50 ng/ml) apoptosis was determined using a CDDE (***p ≤ 0.001) and **e**, **g** the status of XIAP, p-p65(Ser536), and p65 monitored by Western-blot analysis. β-actin served as loading control. **h** Parental and IZI-conditioned A375 melanoma cells were treated with IZ1551 (50 ng/ml) or increasing doses of the smac mimetic SM83 (0.1, 1, 10 µM) alone or in combination. After 16 h apoptosis was determined using a CDDE (**p ≤ 0.01) and **i** the status of XIAP monitored by Western-blot analysis. GAPDH served as loading control. **j** For five unstimulated *mut*BRAF melanoma cell lines the relative expression of XIAP was determined by semi-quantitative Western-blot analysis and **k** the apoptotic response to IZI1551 (50 ng/ml) determined after 16 h using a CDDE (***p ≤ 0.001). For CDDE the mean ± SD of three independently performed experiments is shown. WBs represent one out of three independently performed experiments

Given these model predictions, we wanted to verify whether NFκB-driven up-regulation of XIAP plays a major role in conferring TRAIL resistance in IZI1551-conditioned A375 melanoma cells. As predicted, ectopic expression of a non-degradable IκBα (S32/36A)-SR mutant, preventing NFκB activation, was able to partially re-sensitize conditioned cells to IZI1551 (Fig. 5a), and coincided with increased XIAP-depletion (Fig. 5b). An enhanced turnover of XIAP in parental versus conditioned A375 cells was also evident when we monitored loss of endogenous XIAP upon transcriptional inhibition by Actinomycin D (ActD). While XIAP started to vanish after eight hours of ActD treatment in parental A375 cells, it stayed stable at least for 16 h in conditioned cells (Fig. 5c). Intriguingly, transient knock-down of XIAP using RNA interference was able to almost fully re-sensitize conditioned A375 cells to IZI1551, confirming the predictions of the DBN model (Fig. 5d, e).

Most importantly, and without any previous molecular analysis, siRNA-mediated XIAP knock down antagonized its upregulation by NFκB and consequently fully re-sensitized conditioned Malme3M to IZI1551 (Fig. 5f, g), confirming that XIAP might be a key player in conferring TRAIL resistance in *mut*BRAF melanoma cells, and that the DBN developed here was able to predict this key player correctly. Accordingly, co-treatment with the SMAC mimetic (SM83) was able to re-sensitize IZI1551-conditioned melanoma cells through depletion of XIAP (compare Fig. 5h, i).

To investigate whether XIAP expression level might serve as a biomarker predicting responsiveness to TRAIL receptor-activating agonistic molecules in general, we correlated semi-quantitative XIAP protein expression level with IZI1551 responsiveness in five different *mut*BRAF melanoma cell lines. Strikingly, only cell lines expressing very low XIAP levels, Skmel1, WM35 and WM115, induced pronounced apoptosis in response to IZI1551 treatment, whereas Malme3M and Skmel5, expressing elevated XIAP protein level, only moderately underwent apoptotic cell death (compare Fig. 5j, k).

In summary, we have identified hexavalent TRAIL receptor agonist IZI1551 to be superior in actively inducing cell death in *mut*BRAF melanoma compared to conventional trimeric TRAIL but also compared to specific targeted *mut*BRAF kinase inhibitors as used in the clinic. Above this, we have established a regularized DBN model that was able to predict key players of TRAIL susceptibility correctly and helped to identify XIAP to serve as a potential biomarker for TRAIL treatment responsiveness in *mut*BRAF melanoma.

## Discussion

Defensive mechanisms against cell death render melanoma resistant to current therapeutic outlines with targeted kinase inhibitors.^4^ The molecular mechanism leading to intrinsic or acquired resistance against BRAF-inhibitors is still controversial since pro-apoptotic and anti-apoptotic functions depend on cellular context, target proteins, and cross-talk of different pathways.^7,26,27^ Accordingly, neither treatment of two *mut*BRAF melanoma cell lines with the *mut*BRAF inhibitor dabrafenib alone nor in combination with the MEK inhibitor trametinib yielded significant cell death. In contrast, we demonstrated that the TRAIL receptor agonist IZI1551 potently induced cell death in parental *mut*BRAF as well as dabrafenib-conditioned *mut*BRAF melanoma cells lines, while sparing untransformed primary cells of the skin. It therefore appears that active and tumor-selective induction of apoptosis through death receptor activation might be a promising first or second line treatment alternative for *mut*BRAF melanoma, administered either alone or in combination with targeted *mut*BRAF inhibitors.

However, different mechanisms of intrinsic TRAIL resistance have also been observed in cancer cells, especially in melanoma.^10,28^ IZI1551-specific acquired resistance coincided with two major features, namely down-regulation of the initiator caspase-8 which is indispensable for downstream execution of apoptotic processes, and constitutive activation of the anti-apoptotic transcription factor NFκB. Low caspase-8 levels have been reported to form non-functional heterodimers with the FLICE inhibitory protein (FLIP) that are more stable than the functional caspase-8 homodimers and may lead to NFκB activation instead of cell death induction.^29,30^ In turn, FLIP is transcriptionally regulated by several transcription factors, including NFκB and its expression has been correlated to drug resistance in a wide range of human malignancies.^15,31,32^ Thus, low caspase-8 level together with FLIP may shift the balance at the TRAIL receptors from pro-apoptotic signalling to anti-apoptotic signal transduction via NFκB activation. It is known that constitutive NFκB activation is linked to tumor maintenance and drug resistance.^24,32–34^ Studies investigating the role of NFκB in tumor pathogenesis and the mechanisms regulating its activity, revealed that multiple factors are involved in anti-apoptotic responses, and a better understanding of the molecular mechanisms could lead to new targets identification and prognostic biomarkers.^35^ Accordingly, the canonical NFκB signaling pathway was included into a DBN modelling approach aiming to identify the key differences within the signal transduction networks of parental IZI1551-sensitive versus conditioned IZI1551-resistant *mut*BRAF melanoma cell lines and the molecular mechanism leading to acquired TRAIL resistance.

DBNs as well as the related probabilistic Boolean networks (PBNs) are specifically suited to quantitatively model large-scale regulatory and signalling networks based on steady-state expression or activity data.^17,36^ Based on a minimal parametrization (one parameter per interaction) and a relatively simple algebraic formalism, they obtain superior speed over more detailed kinetic models (e.g., ODE based) while still preserving a good predictive power.^17,37^ The continuous variables of the DBNs/PBNs thereby allow quantitative modelling and predictions in contrast to the classical Boolean approaches with only qualitative read-out. In this study we combined DBN modelling with a sophisticated regularization strategy aiming for sparse and context-sensitive networks and show the performance of this strategy in the detection of cell line-specific deregulations of a signalling network. For ODE based models, the L1 regularization can be used to demonstrate the connections between the deregulation of signal transduction networks and the pathophysiology in cancers.^38,39^ In contrast to their single regularization, our method includes both cell type comparisons and network pruning as part of the overall optimization problem in the form of regularization functions, therefore providing a more stable solution than methods based on independent optimization and unsupervised clustering or multi-step model selection methods. Combining both selection methods resulted in a total of 676 model variants which could efficiently be scanned with the very fast DBN implementation in the FALCON toolbox. This resulted in the simultaneous network pruning, contextualization, and parameter fitting which are tasks which usually are only performed sequentially in other modelling frameworks.

Analysis of the regularized model revealed that a subset of reactions, mainly linked to NFκB and anti-apoptotic signalling, were strongly upregulated in conditioned IZI1551-resistant cells, whereas the essential nodes in the parental cell lines were identified to be mainly pro-apoptotic proteins. The model accurately predicted IκB –SR to partially and XIAP knockout to fully re-sensitize conditioned cells. The continuous regularization paths within the innovative strategy^16^ make sure that the top performing models located in the same region in the BIC landscape will have a similar parametrization and thus yield similar predictions.

Following these predictions, we confirmed that NFκB inhibition by ectopic expression of IκBα-SR mutant partially reduced cellular XIAP levels in conditioned melanoma cells coinciding with partial re-sensitization to IZI1551. More importantly, direct depletion of anti-apoptotic XIAP fully rescued the TRAIL-resistant phenotype, not only in the A375 cell line used for model parameterization, but also in another *mut*BRAF cell line, Malme3M. XIAP, an NFκB-dependent member of the inhibitor of apoptosis (IAP) family, inhibits apoptotic cell death through binding to the executioner caspase-3, −7, and-9, and has been shown to be upregulated in many human tumors.^40–42^ Conversely, XIAP was shown to enhance NFκB activation constituting a positive feedback loop to prevent apoptosis.^9,43,44^ Accordingly, co-application of XIAP-inhibiting SMAC mimetics has successfully been used to sensitize different TRAIL-resistant tumor cells^45,46^ to apoptotic cell death. To that effect, we showed co-stimulation with the SMAC mimetic SM83 to fully re-sensitize melanoma cells to IZI1551 that had acquired secondary resistance to the TRAIL-agonist via XIAP depletion.

Conclusively it turned out that the DBN model combined with the regularization strategy accurately predicted XIAP to be the key player in conferring TRAIL resistance. This became even more evident when we could correlate elevated XIAP expression level in melanoma cells to intrinsic TRAIL resistance, indicating that XIAP may serve as a biomarker for TRAIL responsiveness of *mut*BRAF melanoma. Here, we provide evidence that alterations in the abundance of the NFκB and XIAP proteins change the sensitivity of resistant melanoma cells to IZI1551 treatment. Our studies show that the resistance mechanism is conserved between A375 and Malme3M cells.

Taken together, these results indicate that essential network and cell type-specific reactions can be identified using protein measurements and regularized optimization of a DBN model. The underlying mechanism involved upregulation of NFκB, and predictions identified XIAP as the key player of TRAIL (IZI1551) resistance. Based on the fact that numerous SMAC mimetics are already used in clinical trials for numerous cancers, including leukemia, lymphoma, and solid tumors as single agents or combination therapies,^47^ one could envisage SM83-IZI1551 combinations for the treatment of kinase-inhibitor resistant melanoma patients in the future.

Importantly, incorporation of multiple regularization functions in optimization problems, including logical networks modelling, may provide a promising avenue for future studies to assess the effects of drug combinations and eventually to identify responders to selected combination therapies in a personalized approach.

## Methods

Unless stated otherwise, results of Cell Death Detection ELISA and flow cytometry analysis are presented as mean ± SD of three independently performed experiments. Western-blot analyses represent one out of three independently performed experiments. Statistical analysis of biochemical data was performed using Student’s *t*-test.

### Cells and reagents

Human melanoma cell lines (A375, Malme3M, WM1366, WM1346, Skmel5, Skmel1, WM35, WM115), were maintained in RPMI 1640 medium (Gibco, #61870-010) with 10% FCS (Gibco, #10270-106) in a humified atmosphere of 5% CO_2_ at 37 °C. A375, Malme3M, WM1366, and WM1346 were conditioned to 5 ng/ml IZI1551, A375, and Malme3M to 1 µM dabrafenib over a period of 6 months, adding fresh compound every other day. Primary cells were purchased from Cell Systems and used at passage 4. Keratinocytes (#FC-0007) were maintained in Dermalife®K Complete Medium (Cell Systems, #LN-0027), fibroblasts (#FC-0001) in DMEM (Gibco, #41965-039) and melanocytes (#FC-0030) in Melanocyte Growth Medium (M2, Promocell, #C-24300). For cell death induction 50 ng/ml IZI1551 (University of Stuttgart), 30 µM Cisplatin (TEVA-Deutschland, #2615.03.01), 1 µM Actinomycin-D (Sigma, #A1410), 0.1-1-10 µM SM83 (Baliopharm), 10 µM dabrafenib, or 1 µM trametinib (both Selleckchem, #S2807 and # S2673) was added to cells.

### Plasmids, cloning, and siRNA transfection

For transient expression of IκBα-SR-S32/36A, 6 × 10^6^ A375 cells were electroporated with 20 µg of the plasmid pBK-CMV-IκBα-SR or the empty pBK-CMV vector and investigated 24 h later.

Gene silencing was facilitated by transfecting 5 × 10^4^ cells with 40 pmol siRNA for XIAP- 5′-CGAGCAGGGUUUCUUUAUATT-3′ (Ambion, #AM51331), or lacZ-5′-GCGGCUGCCGGAAUUUACCTT-3′ (MWG Eurofins) using Lipofectamine 2000 (Thermo Scientific, #11668019), 72 h prior to stimulation.

### 3D melanoma spheroids

Melanoma spheroids were generated using the “hanging drop” method.^19^ Briefly, 5 × 10^4^ GFP-expressing melanoma cells were resuspended in 5 ml of medium containing 20% methyl cellulose (Sigma, #M0512). Forty drops of 25 µl containing 250 cells were spotted on the lid of a 10 cm cell culture dish and incubated for 14 days at 37 °C, 5% CO_2_. For in vitro stimulation 160 melanoma spheroids were collected in a 2 cm culture dish previously coated with 1% agarose.

### Flow cytometry

5 × 10^5^ cells were blocked in PBS/2% BSA for 30 min, and incubated with the primary antibodies against TRAIL receptors R1, R2, R3, R4 (huTRAILR1-M271, huTRAILR2-M413. huTRAILR3-M430, huTRAILR4-M444, Amgen) at 2.5 µg/ml in PBS/2% BSA, for 1 h on ice. After washing twice with PBS/1% BSA, 2 µg/ml of the secondary goat-anti-mouse-488 antibody (Thermo Scientific #A-11001, RRID: AB_2534069) in PBS/2% BSA were added for 30 min at 4 °C. Subsequently, cells were washed twice with PBS/2% BSA and subjected to FACS analysis (LSR II, Becton Dickinson). Excitation wavelength used was 488 nm, the emitted green fluorescence (lmax 520 nm) was detected using (FL1) band-pass filter.

### Determination of cell death and clonogenic outgrowth

Apoptosis was determined in a Cell Death Detection ELISA (CDDE, Roche, #11920685001) according to the manufacturer’s protocol. The enrichment of mononucleosomes and oligonucleosomes released into the cytosol is calculated: absorbance of samples/absorbance of control cells at 450 nm (Tecan M200). An enrichment factor of 2 corresponds to 10% apoptosis as determined by AnnexinV-FITC/PI FACS analysis (FACSAria III, Becton Dickinson). For clonogenic assay, 2 × 10^4^ Malme3M or 8.5 × 10^2^ A375 cells were seeded into six-well plates for 8 days or until control cells had reached confluency. Subsequently, cells were stained with crystal violet (0.1 w/v in 20 % Methanol) for 15 min at RT. Cells were washed and crystal violet dissolved from cells with 0.1 M KH_2_PO_4_/EtOH for 5 min at RT and color intensity of supernatants measured at 595 nm (Tecan M200).

### Western-blot analysis

Cells were lysed in lysis buffer (50 mM HEPES, pH 7.5; 150 mM NaCl; 10% glycerol; 1% Triton-X-100; 1.5 mM MgCl_2_; 1 mM EGTA; 100 mM NaF; 10 mM pyrophosphate, 0.01% NaN_3_, phosSTOP^®^ and Complete^®^). After centrifugation, supernatants were collected and the protein content determined by DC Protein assay kit (BioRad). Sixty to 80 µg of protein extracts were subjected to 4–15% gradient SDS-PAGE (BioRad), blotted onto nitrocellulose membranes and incubated with antibodies directed against PARP, XIAP (BD-Biosciences; #551025, RRID:AB_394009; #610717, RRID:AB_398040), caspase-3, IκBα, NFκB-p65, p-p65(S536), AKT, p-AKT(S473), ERK1/2, p-ERK1/2(T202/Y204) (Cell Signaling; #9665, RRID:AB_2069872; #4814, RRID:AB_390781; #8242, RRID:AB_10859369; #3033, RRID:AB_331284; #2920, RRID:AB_1147620; #4060, RRID:AB_2315049; #9102, RRID:AB_330744; #4376, RRID:AB_331772), FLIP, (Sigma #PRS2437, RRID:AB_259702), and caspase-8 (Adipogen #AG-20B-0057, RRID:AB_2490271), respectively. Equal loading was monitored by re-probing membranes with antibodies against GADPH (Cell Signaling #2118, RRID:AB_561053), α-tubulin (Thermo Scientific #MS-581-P1, RRID:AB_144075), or β-actin (Cell Signaling #4970, RRID:AB_2223172). HRP-conjugated secondary antibodies were purchased from GE-Healthcare (Anti-mouse-HRP, RRID:AB_772210; Anti-rabbit-HRP, RRID:AB_772206). Bands were visualized by applying chemiluminescense SuperSignal^®^ detection systems (Thermo Scientific, #34087 and #34076). Protein expression was determined by calculating the ratio between the protein intensities and either α-tubulin, β-actin, or GADPH as housekeeping proteins using ImageQuant 5.2 software (GE Healthcare). Blots derive from the same experiment and have been processed in parallel.

### Mathematical modeling

Quantitative protein expression values as determined by Western-blot analysis were then normalized across all cell lines, experimental conditions, and time-points, to the [0–1] interval, independently for each protein, and the average and standard error of five replicates was calculated. Bayesian modelling was performed using these averaged normalized relative expression values as input.

We generated a DBN, a type of probabilistic logical network model of the main pathways hypothesized to play a role in apoptosis resistance from literature. In this type of network model, nodes representing the relative activity of signalling molecules are linked by simple logical functions. These functions can perform the basic AND, OR, and NOT operations, and be parametrized with proportionality constants. Contextualized network models include parameter values (*k*) for each edge, representing the relative strength of this edge. In contrast with strictly Boolean models, which are qualitative, probabilistic logical models are thus able to provide quantitative estimates. Their formulation however is not as mathematically complex as Ordinary Differential Equations, which are classically used for pharmacological models, therefore their computational cost remains low.

We used the Matlab toolbox FALCON^17^ to contextualize this network with the protein measurements. Briefly, FALCON uses gradient descent to optimize the set of parameter values minimizing the mean of squared error (MSE) between the simulated node intensities of a DBN^46,47^ and the corresponding measured normalized protein expressions. After fitting this network model on steady-state protein measurements, quantitative information can be retrieved, informing on both the relative activity of the signalling molecules in the different experimental conditions, and the strength of interactions between molecules.

To select for context-specific interactions, we optimized the network for both parental and IZI-conditioned cell lines in a single optimization, and included in the objective function two regularization terms to materialize cross-context modelling assumptions. Firstly, we used a fractional norm on parameter values in an effort to prune the network from interactions not strictly necessary to fit the dataset in neither of the cell lines. The use of a fractional norm is dictated by the probabilistic nature of the modelling framework: for each node, the sum of incoming edge strengths must be equal to exactly 1, which renders the L1-norm ineffective to induce sparsity. Secondly, we used a group-level L1-norm across cellular contexts,^48^ using a strategy similar to Merkle et al.^38^ The optimal parameter set *K* of *P* parameters *k*_1_, *k*_2_, *k*_P_ was recovered using the objective function:

1 $$_{K \in \left[ {0,1} \right]^P}^{\arg \,\min }\left( {\frac{1}{N}\mathop {\sum}\limits_{i = 1}^N {\left( {X_i} - \hat{X}_{i} \right)^2 + {\mathrm{\lambda }}_{\mathrm{1}}\mathop {\sum}\limits_{k = 1}^P {\sqrt k + } } {\mathrm{\lambda }}_{\mathrm{2}}\mathop {\sum}\limits_{g = 1}^P {\mathop {\sum}\limits_{j = 1}^{J^g} {\left| {k_j^g - \overline {k^g} } \right|} } } \right)$$

with *N* the number of individual data points in dataset *X* and /hat{*X*} being the set of corresponding values in the simulated network model. The first term is the mean squared error (MSE), the second term is the Lq semi-norm with q = 0.5, while the third term is the grouped L1-norm, with J^g^ the number of members in group g, and /bar{k^g^} the mean value for parameter k in group g. The scalars lambda1 and lambda2 are tuning hyper-parameters controlling the regularization strengths for each of the regularization objectives.

During the systematic knockout experiments, we evaluated models using the Akaike Information Criteria (AIC), which balances goodness-of-fit with model size. AIC is calculated as: Nlog(MSE) + 2*P*.

We recovered the MSE and optimal parameter sets for 676 combinations of lambda1 and lambda2 values (screening each one from 2^−10^ to 2^2^ by half-log steps), and computed for each of these the Bayesian Information Criterion (BIC) as Nlog(MSE) + log(*N*)*P*, and the topology of the optimal network, using a minimal threshold of 0.01 for keeping edges and their corresponding parameters in the model.

## Electronic supplementary material

Supplemental Information

## Data Availability

The dataset generated and analyzed during the current study and the model topology including the scripts are available in the GitHub repository, https://github.com/sysbiolux/FALCON/tree/master/FALCON/ExampleDatasets/DelMistro2018. All other relevant data are available from the authors.

## Electronic supplementary material

**Supplementary information** accompanies the paper on the *npj Systems Biology and Applications* website (10.1038/s41540-018-0075-y).
