# Supplementary material for: Systemic network analysis identifies XIAP and IκBα as potential drug targets in TRAIL resistant BRAF mutated melanoma
Source: NPJ Syst Biol Appl. 2018 Nov 5;4:39. doi: 10.1038/s41540-018-0075-y (PMC6218484; doi:10.1038/s41540-018-0075-y)
Supplement: Supplementary file 1 — Supplemental Information [file 41540_2018_75_MOESM1_ESM.pdf]

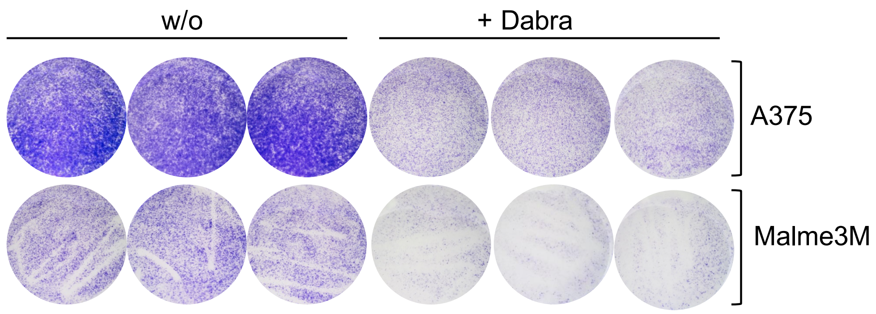

Figure S1: Clonogenic outgrowth of *mut*BRAF melanoma cells in response to dabrafenib. $8.5x10^2$ A375 or $2x10^4$ Malme3M cells were seeded into 6-well plates and stimulated or not with dabrafenib (Dabra, 10 µM) for 8 days. Subsequently, cells were stained with crystal violet. Three independently performed experiments per cell lines are shown.

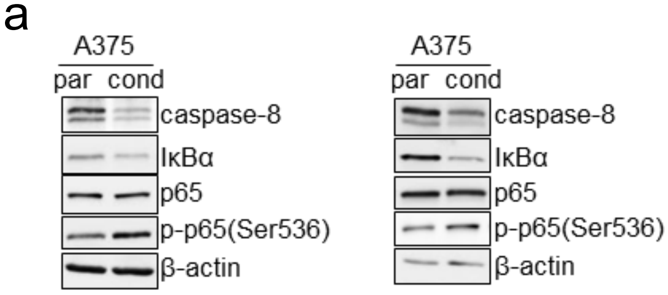

b

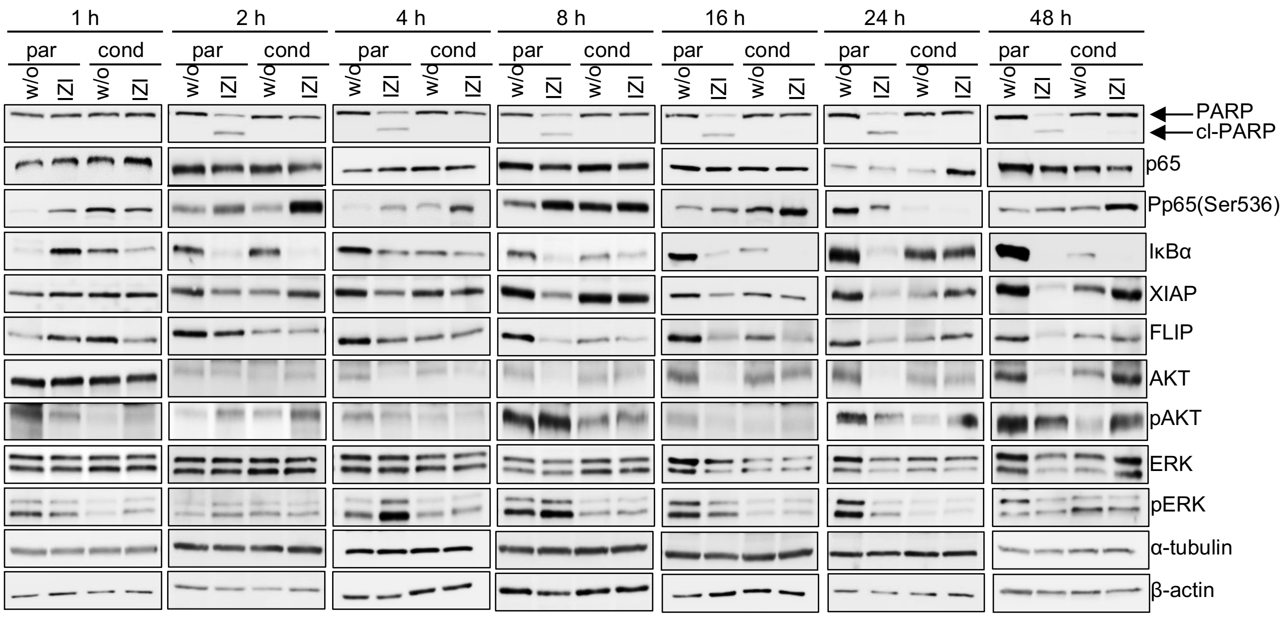
1 h
2 h
4 h
8 h
16 h
24 h
48 h
par
cond
w/o
IZI
PARP
cl-PARP
p65
Pp65(Ser536)
IκBα
XIAP
FLIP
AKT
pAKT
ERK
pERK
α-tubulin
β-actin

c

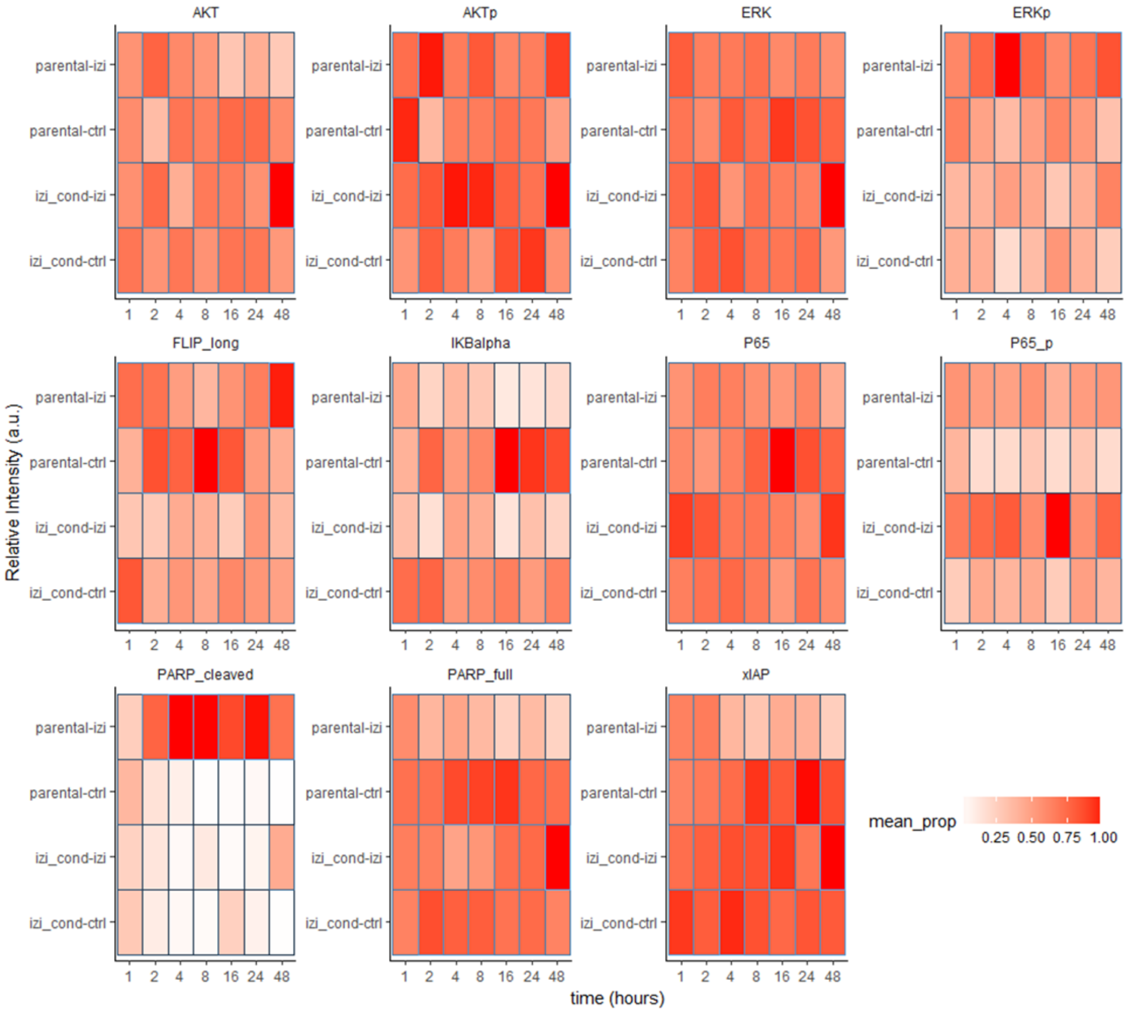
AKT
AKTp
ERK
ERKp
FLIP_long
IKBalpha
P65
P65_p
PARP_cleaved
PARP_full
xIAP
parental-izi
parental-ctrl
izi_cond-izi
izi_cond-ctrl
1 2 4 8 16 24 48
Relative Intensity (a.u.)
time (hours)
mean_prop
0.25 0.50 0.75 1.00

Figure S2: Protein expression pattern of TRAIL-sensitive parental versus TRAIL-resistant conditioned melanoma cells. (a) The expression level of caspase-8, IκBα, NFκB(p65) and phosphorylated-p65(Ser536) in untreated parental and IZI-conditioned A375 cells was monitored by Western-blot analysis. β-actin served as loading control **(b)** Expression and/or activation of PARP, NFκB(p65), pp65(Ser536), IκBα, XIAP, FLIP, AKT, pAKT, ERK, pERK was assessed in *mut*BRAF A375 melanoma cell lines at the indicated time points be Western-blot analysis. α-tubulin and β-actin served as loading control. One representative out of 5 independently performed experiments is shown. **(c)** Normalized time course Western-blot data comparing parental and IZI conditioned A375 cells upon IZI1551 treatment. The represented heatmaps show the mean expression of 5 replicates with the highest intensity scaled to 1.

	target	dose [ng/ml]	treatment	scaled_mean (%apoptosis)	scaled_error
1	apoptosis	0	ctrl	5.0	0
2	apoptosis	0.5	IZI	17.0	2.2
3	apoptosis	2	IZI	25.6	1.1
4	apoptosis	4	IZI	33.9	3.4
5	apoptosis	6	IZI	40.3	1.6
6	apoptosis	8	IZI	41.6	6.6
7	apoptosis	10	IZI	58.6	8.6
8	apoptosis	25	IZI	61.1	5.13
9	apoptosis	50	IZI	64.5	6.8

Table S1: IZI dose response data used for the EC50 calculation.

parameter values			
**reaction**	**parental**	**conditioned**	**interpretation**
BCL2-|Casp3cl	0.013	0.013	equal
Casp3cl->Apoptosis	0.500	0.501	equal
Casp3cl-|XIAP	0.379	0.004	different
Casp8cl-|cFLIP	0.354	0.365	equal
cFLIP->pAKT	0.981	0.984	equal
cFLIP->pIKK	0.071	0.192	different
cFLIP->pERK	0.736	0.736	equal
cFLIP-|Casp8cl	0.006	0.005	equal to 0
FADD->pIKK	0.921	0.795	different
housekeeper->cFLIP	0.679	0.680	equal
housekeeper->pp65	0.004	0.008	equal to 0
housekeeper->XIAP	0.731	0.730	equal
housekeeper_BRAF->BRAF	0.022	0.021	equal
IKBalpha-|pp65	0.006	0.006	equal to 0
NRAS->pAKT	0.019	0.016	equal
NRAS->BRAF	0.978	0.979	equal
pAKT->pIKK	0.008	0.013	equal
pAKT->Survival	0.012	0.020	equal
pAKT-|BRAF	0.005	0.005	equal to 0
PARPcl->Apoptosis	0.500	0.499	equal
pERK->pp65	0.473	0.118	different
pERK->Survival	0.963	0.954	equal
pIKK-|IKBalpha	0.850	0.859	equal
pIKK->pp65	0.523	0.874	different
pMEK->pERK	0.264	0.264	equal
pp65->cFLIP	0.321	0.320	equal
pp65->Survival	0.025	0.026	equal
pp65->XIAP	0.269	0.270	equal
XIAP-|Casp3cl	0.013	0.871	different

Table S2: Table comparing the optimal parameter values used for model predictions after L1/2 and L1 groups regularization.
